# Early outcomes of kidney transplantation from elderly donors after circulatory death (GEODAS study)

**DOI:** 10.1186/s12882-019-1412-0

**Published:** 2019-06-26

**Authors:** María José Pérez-Sáez, Omar Lafuente Covarrubias, Domingo Hernández, Francesc Moreso, Edoardo Melilli, Javier Juega, Erika de Sousa, Paula López-Sánchez, María Luisa Rodríguez-Ferrero, Naroa Maruri-Kareaga, María Dolores Navarro, Rosalía Valero, María Auxiliadora Mazuecos, Francisco Llamas, Paloma Martín-Moreno, Antón Fernández-García, Jordi Espí, Carlos Jiménez, Ana Ramos, Eva Gavela, Julio Pascual, Jose M. Portolés, on behalf of the GEODAS Group, José M. Portolés, Omar Lafuente Covarrubias, Paula López-Sánchez, Beatriz Sánchez-Sobrino, María José Pérez-Sáez, Julio Pascual Santos, Ana Zapatero, Domingo Hernández-Marrero, Francesc Moreso, Edoardo Melilli, Anna Manonelles, Javier Juega, Ricardo Lauzurica, Erika de Souza, Fritz Diekmann, María Luisa Rodríguez-Ferrero, Naroa Maruri-Kareaga, Sofia Zárraga, María Dolores Navarro, Alberto Rodríguez-Benot, Rosalía Valero, Juan Carlos Ruiz, María Auxiliadora Mazuecos, Francisco Llamas, Paloma Martín-Moreno, Ángel Alonso, Antón Fernández-García, Jordi Espí, Isabel Beneyto, Carlos Jiménez, María López-Oliva, Ana Ramos, Eva Gavela, Asunción Sancho-Calabuig

**Affiliations:** 1 Nephrology Department and Kidney Transplantation Program, Hospital del Mar, Parc de Salut Mar, Universitat Autónoma Barcelona and Universitat Pompeu Fabra, Nephropaties Research Group Institute Mar for Medical Research, Passeig Maritim 25-29, 08003 Barcelona, Spain; 2 Nephrology & Transplant Department, University Hospital Puerta de Hierro, Universidad Autónoma Madrid, Public Research Net RedInRen ISCIII 016/009, C/Manuel de Falla s/n, 28222 Majadahonda, Madrid Spain; 3 H.U. Carlos Haya, Malaga, Spain; 4 H. Vall d’Hebron, Barcelona, Spain; 5 H. Bellvitge, Barcelona, Spain; 6 H. German Trias y Pujol, Barcelona, Spain; 7 H. Clinic de Barcelona, Barcelona, Spain; 8 HGU Gregorio Marañón, Madrid, Spain; 9 H.U. Cruces, Bilbao, Spain; 10 H.U. Reina Sofia, Córdoba, Spain; 11 H.U. Marqués de Valdecilla, Santander, Spain; 12 H.U. Puerta del Mar, Cádiz, Spain; 13 H.U. de Albacete, Albacete, Spain; 14 C.U de Navarra, Pamplona, Spain; 15 C.H.U de A Coruña, A Coruña, Spain; 16 H.U. La Fe, Valencia, Spain; 17 H.U. de la Paz, Madrid, Spain; 18 F. Jiménez Díaz, Madrid, Spain; 19 H.U. Dr Peset, Valencia, Spain

**Keywords:** Kidney transplantation, Elderly donors, Donors after circulatory death, Clinical outcomes, Delayed graft function

## Abstract

**Background:**

Spain has dramatically increased the number of controlled circulatory death donors (cDCD). The initial selection criteria for considering cDCD for kidney transplantation (KT) have been expanded progressively, with practically no limits in donor age during the last years. We aimed to analyze the early clinical outcomes using expanded (> 65 years) cDCD in comparison with standard ones.

**Methods:**

Observational multicenter study including 19 transplant centers in Spain. We performed a systematic inclusion in a central database of every KT from expanded cDCD at each participant unit from January-2012 to January-2017. Surgical procedures and immunosuppressive protocols were based on local practices. Data was analyzed in the central office using logistic and Cox regression or competitive-risk models for multivariate analysis. Median time of follow-up was 18.1 months.

**Results:**

561 KT were performed with kidneys from cDCD, 135 from donors older than 65 years. As expected, recipients from older cDCD were also older (65.8 (SD 8.8) vs 53.7 (SD 11.4) years; *p* < 0.001) and with higher comorbidity. At 1 year, no differences were found amongst older and younger cDCD KT recipients in terms of serum creatinine (1.6 (SD 0.7) vs 1.5 (SD 0.8) mg/dl; *p* = 0.29). Non-death censored graft survival was inferior, but death-censored graft survival was not different (95.5 vs 98.2% respectively; *p* = 0.481). They also presented a trend towards higher delayed graft function (55.4 vs 46.7%; *p* = 0.09) but a similar rate of primary non-function (3.7 vs 3.1%; *p* = 0.71), and acute rejection (3.0 vs 6.3%; *p* = 0.135). In the multivariate analysis, in short follow-up, donor age was not related with worse survival or poor kidney function (eGFR < 30 ml/min).

**Conclusions:**

The use of kidneys from expanded cDCD is increasing for older and comorbid patients. Short-term graft outcomes are similar for expanded and standard cDCD, so they constitute a good-enough source of kidneys to improve the options of KT wait-listed patients.

## Background

Kidney transplantation (KT) from donors after cardiac death (DCD) might imply poorer graft outcomes, as circulatory death constitutes an injury to the organs that may result in lower graft survival [1]. To minimize that, DCD are only considered for KT if they fit strict criteria, including younger donor age than donors after brain death (DBD). In the US, around 50% of expanded criteria donor (ECD)-DCD kidneys are discarded, compared to 30–40% of ECD-DBD [2]. However, a significant number of discarded ECD-DCD kidneys may be acceptable for KT [2]. In fact, as age of patients listed for KT is continuously rising [3, 4], DCD age criteria has also increased in recent years in many countries, especially in Europe [5–11], where policies are more open to expand donor pool criteria, in contrast to the US, where the potential poorer outcomes could lead to a higher discarded organ rate.

In Spain, improvements in organ procurement and assessment tools have allowed us to expand donor acceptance criteria [12]. Particularly, age limits have been expanding, so that age itself is not usually a significant limiting factor. In contrast, more than half of available kidneys from donors ≥65 years old are discarded in the US [13], despite their argued benefits in terms of patient survival, both after brain death [14–16] and after circulatory death [17], comparing to remain on dialysis. However, the increase in donor age is associated with reduced graft function as well as limited recipient and graft survival [18]. Moreover, it has been recently postulated that the use of aged DCD could be detrimental in elderly recipients [10, 11].

In 2012, Spain developed a strategy to encourage the use of controlled DCD (cDCD) for transplantation, a modality less used in our environment because of the high rate of brain-death donation [19]. That resulted in a dramatic increase in cDCD transplantation, from 1.4% of the total donors in 2012 to 18% in 2016 [20]. The same year, GEODAS working group was created with the purposes of: 1) collecting data regarding KT from cDCD; and 2) sharing protocols, outcomes analysis and experiences [21].

As donor age in this modality has also experienced a substantial change during the last years, we aimed to analyze the early results obtained with KT from elderly cDCD donors, both in terms of early clinical outcomes – primary non function (PNF) and delayed graft function (DGF) – as well as 1st year patient and graft survival, in order to reassure with the strategy of using these kidneys for aged recipients or change the policy if needed.

## Methods

Observational and multi-center registry, including 19 transplant centers in Spain. Data from all cDCD (Maastricht type III) KT performed from January 2012 to January 2017 were collected and prospectively recorded by nephrologists at each center database following the same structure. For this analysis, a data manager central office merged anonymous databases in a single one. Informed consent for kidney transplantation and local electronic data management was obtained according to each center’s Institutional Review Board policy, and a central research Ethics Committee of H.U. Puerta de Hierro approved the project. A pre-defined analysis after the first 500 cDCD KT was performed in order to evaluate the early results obtained with kidneys from donors over 65 years, and to establish strategy changes if needed. This age cut-point was chosen based on the upper limit recommended by the 1st version of Spanish National Transplant Organization cDCD strategy guides in 2012 [19]. In Spain, old donors usually allocate into old recipients, but there are not any pre-established common strategy between centers, being the finally decision made by each center. Sample size was estimated for a 15% difference in DGF incidence rate between groups.

Graft extraction was performed with or without ante-mortem vascular cannulation, depending on the center. The immunosuppressive regimen included induction with rabbit anti-thymocyte globulin (Thymoglobulin®, Sanofi, France) or basiliximab (Simulect®, Novartis, Swizerland), and maintenance with steroids as well as the combination tacrolimus-mycophenolate or tacrolimus-everolimus, tailored to patient immunological risk and according to center’s local practice.

Local standardized serum creatinine (colorimetric) and glomerular filtration rate (GRF) estimated by Modified-Diet Renal Disease-4 (MDRD-4) formula were recorded at month 1, 3, 6 and every 6 months thereafter. Clinical events such as acute rejection, graft loss or patient death were recorded as they occurred. Delayed graft function (DGF) was defined as the need of dialysis during the 1st week after KT.

### Statistics

Data are shown as mean and standard deviation or percentages and event rates depending on variable nature. Continuous variables were compared using t-Student statistics or Wilcoxon’s test if variables were not normally distributed. Categorical variables were compared with two-tailed Chi-square statistics. Significance was considered when *p* < 0.05. Patient and graft survival was estimated by Kaplan-Meier curves (log-rank test). Three statistical models for survival were used to increase the robustness of the analysis: logistic regression, Cox regression and competing risk regression analyses.

Logistic regression was used to estimate Odds Ratio (OR) for 1st year cumulative mortality. Uni and multivariate Cox regression models were carried out to estimate hazard ratio (HR) for survival. Backward step multivariable regression was performed with principal variable (old vs. young donor), considering as possible confounders from donor those included in KDPI (ethnicity, cause of death, serum creatinine > 1.5 mg/dl, diabetes mellitus, hypertension, etc.). We also included other potential confounders as recipient age, sex and comorbidity, cold ischemia time (CIT), human leukocyte antigen (HLA) mismatches, prior dialysis modality, prior KT, time on dialysis prior to transplant as well as other usual risk variables such as serum creatinine or induction treatment. Variables with *p* value< 0.1 in the univariate analysis were included in the multivariable backward modeling process, besides other variables that were clinically relevant for the outcome. We also used competitive-risk models for graft and patient survival, considering graft loss, death and loss to follow-up as competitive events. Results are shown as sub-Hazard Ratio (sHR).

On the other hand, we tested multivariate models for the impact of donor age group on early clinical outcomes: primary-non-function (PNF, defined as grafts that never functioned), DGF, poor kidney function at 12 months (defined as eGFR < 30 mL/min). Patients with PNF were excluded for further analyses of other outcomes.

Analysis was performed using Stata v14 (StataCorp 2015, Stata Statistical Software, College Station, TX).

## Results

561 KT recipients from cDCD were included in the study. Of them, 135 received a KT from a cDCD donor > 65 years. Compared to patients that received a KT from a younger donor, patients that received a KT from cDCD > 65 years were older (65.8 (8.8) years vs 53.7 (11.4) years, *p* < 0.001), more frequently females, diabetics (51.4% vs 26.1%, p < 0.001) and with higher percentage of previous cardiovascular events (20.7% vs 7.8%, p < 0.001). Donor cause of death was more frequently a stroke. They were also better HLA matched and received less frequently thymoglobulin as induction therapy (Table 1).

**Table 1:** Baseline characteristics among kidney transplant recipients from donors > 65 years and ≤ 65 years

	Donor ≤65 years (*n* = 426)	Donor > 65 years (*n* = 135)	*p*-value
Recipient characteristics			
Age (years, mean (SD))	53.7 (11.4)	65.8 (8.8)	< 0.001
Age > 65 years (%)	14.6	61.3	< 0.001
Female Gender (%)	30.1	35.6	0.23
Black Race (%)	1.4	0.7	0.5
Diabetes mellitus (%)	26.1	51.4	< 0.001
Previous cardiovascular event^a^ (%)	7.8	20.7	< 0.001
Cause of end-stage renal disease (%)			
*Hypertensive nephropathy*	12.4	11.9	
*Diabetic nephropathy*	11.3	22.2	
*Glomerulonephritis*	19.0	12.6	
*Interstitial*	11.0	11.9	0.03
*Polycystic*	15.3	11.1	
*Others*	6.7	5.9	
*Unknown*	19.7	23.7	
Previous renal replacement therapy (%)			
*Hemodialysis*	73.1	83.0	
*Peritoneal Dialysis*	21.0	14.8	0.05
*Preemptive kidney transplant*	5.9	2.2	
Dialysis vintage (years, median [IQR])	1.1 [2.0–3.6]	1.3 [2.6–4.0]	0.08
Patients with previous kidney transplant (%)	8.4	7.4	0.7
Donor characteristics			
Age (years, mean (SD))	52.7 (9.1)	72.0 (4.9)	< 0.001
Female Gender (%)	26.3	45.9	< 0.001
Expanded criteria donors (%)	29.3	100	< 0.001
Stroke as Cause of death (%)	49.2	67.8	< 0.001
Transplant characteristics			
Number of HLA mismatches (median (IQR))	4 [3–5]	4 [3–5]	0.003
Cold ischemia time (hours, median (IQR))	11 [7–18]	9.5 [7–16.5]	0.326
Warm ischemia time (min, median (IQR))	23.5 [15–36.5]	26 [16–35]	0.901
Induction treatment (Thymoglobulin, %)	70.7	57	0.003
Maintenance (Tacrolimus+MPA + steroids, %)	82.9	80.7	0.57
Time of follow-up (years, median [IQR])	1.6 [0.9–2.6]	1.1 [0.7–1.8]	< 0.001

*SD* standard deviation, *MPA* mycophenolic acid, *IQR* interquartile range

^a^Acute myocardial infarction, stroke or peripheral vascular disease (amputation)

Regarding early clinical events, the group that received a KT from an older cDCD experienced a trend towards higher rate of DGF, though the difference was not statistically significant (55.4% vs 46.7%, *p* = 0.09). Neither we found any difference in PNF or acute rejection rates (Table 2). When analyzing other potential risk factors for PNF and DGF through logistic regression models, CIT longer than 17 h was found to be a risk factor for PNF (OR 3.25 [1.02–10.33; *p* = 0.046]), while dialysis vintage longer than 24 months prior to KT conditioned DGF (OR 3.44 [2.39–4.94; *p* < 0.001, Table 3).

**Table 2:** Patient and transplant outcomes among kidney transplant recipients from donors > 65 years and ≤ 65 years

	Donor ≤65 years (*n* = 426)	Donor > 65 years (*n* = 135)	*p*-value
Early patient mortality (at first year, %)	1.9	6.9	0.004
Primary non-function (%)	3.1	3.7	0.71
Delayed graft function (%)	46.7	55.4	0.09
Acute rejection (%)	6.3	3.0	0.135
Creatinine at 12 month (mg/dl, mean (SD))	1.51 (0.8)	1.60 (0.7)	0.29
eGFR at 12 month (ml/min, mean (SD))	57.9 (24.7)	49.2 (20.0)	< 0.001

*eGFR* estimated glomerular filtration rate calculated by MDRD-4

**Table 3:** Risk factors for relevant patient and allograft early outcomes estimated with multivariate logistic regression analysis

	OR	95% CI	*p*-value
Primary non-function			
Donor age > 65 years (vs ≤65 years)	2.50	[0.77–8.09]	0.128
Cold ischemia time > 17 h (vs shorter)	3.25	[1.02–10.33]	0.046
Delayed graft function			
Donor age > 65 years (vs ≤65 years)	1.33	[0.87–2.03]	0.19
Dialysis vintage > 24 months (vs shorter)	3.44	[2.39–4.94]	< 0.001
eGFR< 30 ml/min at month 12			
Donor age > 65 years (vs ≤65 years)	1.15	[0.60–2.20]	0.427
Delayed graft function (vs immediate)	4.12	[2.07–8.22]	< 0.001
Early patient mortality (first year)			
Donor age > 65 year (vs ≤65 years)	1.37	[0.46–4.10]	0.578
Recipient age (per year)	1.11	[1.04–1.19]	0.002

*OR* odds ratio, *CI* confidence interval, *eGFR* estimated glomerular filtration rate

In terms of graft function, recipients from older donors showed lower eGFR at 1 year, but not statistically different serum creatinine (1.60 vs 1.51 mg/dl; *p* = 0.29, Table 2). In the multivariate analysis, the risk for poor renal function (1st year eGFR < 30 ml/min) associated with DGF (4.12 [2.07–8.22]; p < 0.001) but not with donor age (Table 3).

On the other hand, cumulative mortality during 1st year was higher for recipients of older cDCD grafts (1.9% vs 6.9%; *p* = 0.004, Table 2). Logistic regression model adjusted by recipient age and comorbidities showed that recipient age was the only risk factor related to patient death, conferring a 11% excess risk for mortality per each recipient’s year (OR 1.11 [1.04–1.19]; *p* = 0.002, Table 3).

**Fig. 1:**
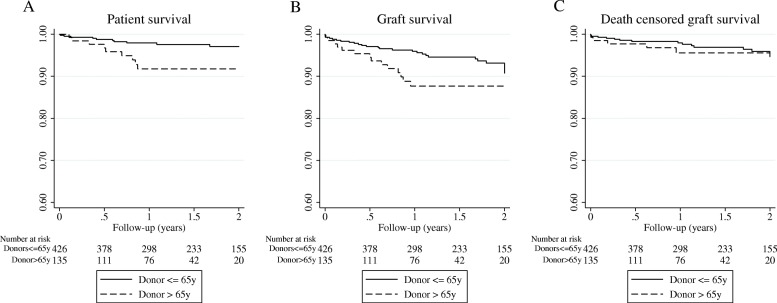
Kaplan-Meier survival curves between kidney transplant recipients from donors > 65 years and ≤ 65 years. **a** Patient survival; **b** Graft survival; **c** Death-censored graft survival

Survival analyses (estimated by KM method) showed a lower 1st-year-graft survival among those kidneys from cDCD donors> 65 years (87.6% vs 96.2%; *p* = 0.02). However, death-censored graft survival was similar in both groups (95.5% vs 98.25; *p* = 0.481) (Fig. 1). We performed Cox regression and competing risk multivariate analyses in order to analyze donor age impact on graft survival. Adjusted-models did not show that donor age itself and isolated was associated with graft survival but eGFR< 30 ml/min at 1-year increased the risk eight -fold (sHR 8.34 [2.82–24.65; p < 0.001, Table 4).

**Table 4:** Risk factors for relevant patient and allograft early outcomes estimated with Cox regression analysis (HR) and competing risk analysis (sHR)

	Risk factor	HR	95% CI	*p*-value	sHR	95% CI	*p*-value
Patient survival							
Model 1 (raw)	Donor age > 65y (vs ≤ 65 years)	2.94	[1.22–7.11]	0.016	3.17	[1.38–7.27]	0.015
Model 2 (adjusted by recipient age)	Donor age > 65y (vs ≤ 65 years)	1.17	[0.44–3.08]	0.751	1.26	[0.48–3.34]	0.636
	Recipient age	1.10	[1.04–1.16]	0.001	1.1	[1.05–1.16]	< 0.001
Model 3 (adjusted by recipient age + previous CV event)	Donor age > 65y (vs ≤ 65 years)	1.06	[0.40–2.82]	0.900	1.06	[0.36–3.12]	0.910
	Recipient age	1.10	[1.04–1.16]	0.001	1.09	[1.04–1.15]	< 0.001
	Previous CV event	2.02	[0.76–5.36]	0.158	2.00	[0.73–5.47]	0.178
Death-censored graft survival							
Model 1 (raw)	Donor age > 65y (vs ≤ 65 years)	1.30	[0.47–3.60]	0.620	1.28	[0.47–3.47]	0.631
Model 2 (adjusted by recipient age)	Donor age > 65y (vs ≤ 65 years)	1.17	[0.44–3.08]	0.751	1.00	[0.30–3.38]	0.997
	Recipient age	1.10	[1.04–1.16]	0.001	1.02	[0.97–1.07]	0.425
Model 3 (adjusted by eGFR< 30 ml/min at first month)	Donor age > 65y (vs ≤ 65 years)	0.66	0.15–3.01	0.595	0.58	[0.12–2.74]	0.487
	eGFR< 30 ml/min at first month	8.70	2.99–25.25	< 0.001	8.34	2.82–24.65	< 0.001

*HR* hazard ratio, *sHR* sub-hazard ratio, *CI* confidence interval, *eGFR* estimated glomerular filtration rate, *CV* cardiovascular

On the other hand, 1st-year patient survival was also poorer (91.8% vs 97.9%; *p* = 0.01). Nine patients from the group of older donors died with functioning graft during the first year after transplantation (mostly in the first 6 months after transplantation) due to cardiovascular events (*n* = 4), sudden death of uncertain origin (*n* = 1), cancer (*n* = 1), sepsis (*n* = 1), pulmonary embolism (*n* = 1), and unknown cause (*n* = 1). Again, the multivariate analysis revealed that only recipient age was as risk factor for patient survival, increasing the risk around 10% per each recipient’s year of age, both with Cox and competing risk analyses (Table 4).

Finally, we aimed to investigate which old donors might carry a higher risk for the recipients, considering early clinical outcomes. By adding classical donor cardiovascular risk factors (serum creatinine > 1.5 mg/dl, diabetes mellitus and cardiovascular as the cause of death), we found that older donors with one or two of those previous risk factors associated with significant higher DGF rate (Table 5). Similar analyses for PNF, poor kidney function (1st year eGFR < 30 ml/min) and 1st year mortality were not significant (data not shown).

**Table 5:** Risk factors for relevant renal allograft outcomes estimated with logistic regression analysis

	OR	95% CI	*p*-value
Delayed graft function			
Donor age > 65 years + 0 risk factor	1.32	0.79–2.22	0.30
Donor age > 65 years + 1 risk factor	1.79	1.05–3.05	0.03
Donor age > 65 years + 2 risk factors	3.84	1.91–7.72	< 0.001

Donor risk factors are defined as creatinine > 1.5 mg/dl, cardiovascular death and hypertension. Similar analyses for Primary non function, estimated glomerular filtration rate < 30 ml/min at 12 months and early patient mortality were not significant

## Discussion

In this study, we present early clinical outcomes using KT from cDCD older than 65 years. We found higher 1st year mortality among KT patients receiving a kidney from an elderly donor, but it seems to be related to recipient age. Donor age itself and isolated did not have any impact on patient neither graft survival at short term, although it was associated with poorer graft function in terms of eGFR.

As ECD-DCD donors may constitute more than 40% of the total DCD donor pool in some centers [11], efforts are targeted now to analyze outcomes using kidneys that come from those suboptimal donors. Some reports have analyzed outcomes using ECD-DCD donors in the US [1, 22, 23], Europe [7–11] and Japan [24]. Overall, results from ECD-DCD donors are poorer than those obtained with standard-DCD donors, though not inferior to ECD-DBD donors [1, 7, 9, 22, 24]. However, these studies included KT recipients from classical ECD defined by Organ Procurement Transplant Network (OPTN, donors older than 60 years-old, or between 50 and 60 years-old with two of the following risk factors: cardiovascular death, serum creatinine > 1.5 mg/dl or hypertension) but few studies have analyzed the results with very elderly donors, i.e., older than 65 years-old [10].

Normally, studies have not been focused on patient survival using expanded DCD donors, though we can find a reported one-year-patient survival ranged from 85 to 91% among recipients who received kidneys from ECD-DCD donors [7, 10, 23]. Two studies also compared the mortality between those who underwent KT from an ECD-DCD donor and those who remained waitlisted on dialysis. They found that among older recipients (> 65 years), there was no benefit in terms of survival using kidneys from old DCD donors (> 65 years) [10, 11], and younger recipients might have the potential benefit of the expansion in DCD donor criteria acceptance [11]. In a recent observational study in patients older than 65 years old receiving an old kidney from DCD donors over 65, Peter-Sengers et al. showed a 15% higher mortality than we found among our recipients. Although we have included recipients younger than 65 years old, our cohort included recipients of a special high-risk profile, with a mean age near 66 years, high prevalence of diabetes (> 50%) and previous cardiovascular events (> 20%). When we analyzed the risk factors implicated in early patient survival, we confirmed that only patient age was involved in patient prognosis without any influence of donor age or donor comorbidity. These findings highlight the importance of a carefully selection of the recipient, in order to avoid early patient mortality.

In terms of graft survival, kidney grafts from ECD-DCD donors have shown inferior survival than those from standard DCD donors [1, 24], though similar to ECD-DBD ones [7, 22]. One-year-graft survival has been reported from 74 to 90% [7, 9, 23, 24], similar to ours, despite our donors were almost 5 years older than those from the study by Peter-Sengers et al. which reported the oldest ones so far [10]. In fact, we found no differences in 1st year death-censored-graft survival regardless donor age, highlighting that differences observed in non-censored graft survival are related to a higher rate of patient mortality during the 1st year within recipients who received a kidney from an older donor.

Disparities have been found in other outcomes such as DGF or PNF. While the US Registry found no differences in terms of among ECD-DCD donors and non-ECD-DCD donors, the UK cohort found a higher rate of both PNF and DGF between older donors but comparing to donors younger than 40 years [7]. However, an update of the UK Registry showed similar rates of PNF and similar 5-year graft survival between ECD-DCD and brain-dead ECD KT [8]. The study from the Netherlands found a high-rate of both PNF (12.7%) and DGF (74.1%) in recipients > 65y who received organs from donors > 65 y [10]. Our PNF rate was similar between the groups and below 4% and the only factor that increased the risk was a prolonged cold ischemia time, consistently with other authors’ findings [7]. In our study, the incidence of DGF was high in both groups, with a clear tendency of higher rate among recipients from older donors. The multivariate analysis showed that donor age itself was not associated with higher rate of DGF, but if we considered high-risk donor profile (older plus other comorbidities) the risk is increased by three-fold.

Poorer kidney function has also been shown in kidneys from ECD-DCD donors [7, 9]. In fact, two-thirds of old recipients from DCD donors over 65 y presented with eGFR< 30 ml/min at 1st year [10]. We did not find any statistical difference in terms of serum creatinine at 1st year after transplantation although kidney function was poorer in recipients from elderly cDCD.

Our study is limited by the sample size and the relative short-term follow-up. However, we aimed to analyze 1st year results using elderly cDCD in our media, to know results and implement the proper strategy changes if needed. In fact, this is the first study that analyzes outcomes in an important number of elderly cDCD (mean age 72 years) allocated to a high-risk recipient cohort. We found a higher mortality compared to recipients who received organs from younger donors. However, in the multivariate model the recipient age accounted for all the risk and the weight of the donor age over the death risk disappeared. Three different multivariate statistical models showed these results, which reflects their consistency. On the other hand, short-term graft survival was similar and donor age itself did not have any impact in patient or graft outcomes.

## Conclusions

Our results pointed at similar patient and KT short-term outcomes regardless of donor age, which may suggest better results than those previously reported. If these findings were confirmed in long-term studies, they might generate changes in acceptance and allocation of these grafts for KT. In fact, when we added comorbidity factors other than donors’ age, some outcomes as DGF seem to be affected. Careful pre-transplant evaluation should be performed in these extremely high-risk group in order to improve outcomes, but kidneys from old cDCD should not be discarded systematically.

## Data Availability

The database and the analysis are safeguarded in the central servers of a public health institution (Hospital Puerta de Hierro). Any access to the original database should be properly requested to the corresponding author and approved by Ethics Scientific Committee.

## Abbreviations

cDCD: Controlled donor after circulatory dead
CIT: Cold ischemia time
DBD: Donor after brain death
DCD: Donor after circulatory death
DGF: Delayed graft function
ECD: Expanded criteria donor
eGFR: Estimated glomerular filtration rate
GEODAS (from the words in Spanish): Grupo Español de Trasplante renal con programas de Donante en Asistolia tipo 3 (Spanish Group with cDCD KidneyTransplant Programs)
HLA: Human leucocyte antibody
KT: Kidney transplant
MDRD: Modified-diet renal disease
PNF: Primary non-function
